# The role of comprehensive nursing intervention in postoperative recovery of gastric cancer patients: a retrospective historical cohort study

**DOI:** 10.3389/fmed.2026.1876528

**Published:** 2026-08-11

**Authors:** Hao Wu, Ling Wang, Yu Zhang

**Affiliations:** 1 Department of Gastrointestinal Surgery, The First People’s Hospital of Jiangxia District, Hubei University of Medicine, Wuhan, Hubei, China; 2 Department of Oncology, The First People’s Hospital of Jiangxia District, Hubei University of Medicine, Wuhan, Hubei, China; 3 Department of Operating Room, The First People’s Hospital of Jiangxia District, Hubei University of Medicine, Wuhan, Hubei, China

**Keywords:** enhanced recovery after surgery, gastrectomy, gastric cancer, nursing intervention, perioperative care, postoperative recovery

## Abstract

**Background:**

Gastric cancer remains a significant global health burden, with surgical resection serving as the primary treatment modality. Postoperative recovery is often complicated by gastrointestinal dysfunction, nutritional deterioration, and psychological distress. The effectiveness of comprehensive nursing interventions based on enhanced recovery after surgery (ERAS) principles in Chinese gastric cancer patients remains inadequately investigated.

**Objective:**

To evaluate the effectiveness of a comprehensive nursing intervention program on postoperative recovery outcomes, nutritional status, and psychological well-being in patients undergoing radical gastrectomy for gastric cancer.

**Methods:**

This retrospective, single-centre historical cohort study included 178 patients who underwent radical gastrectomy for gastric cancer at a tertiary hospital. The control group (*n* = 86) received conventional perioperative nursing care during 2022, whereas the intervention group (*n* = 92) received a comprehensive ERAS-based nursing intervention during 2023–2024. Because the two groups were treated in different calendar periods, the design is regarded as a historical cohort study and is reported in accordance with the STROBE guideline. Primary outcomes were postoperative hospital stay and complication rate; secondary outcomes were gastrointestinal recovery, nutritional and psychological indicators, pain, and satisfaction. In addition to unadjusted comparisons, multivariable regression, 1:1 propensity-score matching, and inverse-probability-of-treatment weighting were used to adjust for baseline confounders including sex, and the Benjamini-Hochberg false-discovery-rate procedure was applied to secondary outcomes.

**Results:**

The intervention group demonstrated significantly shorter postoperative hospital stay (7.42 ± 1.44 vs. 10.56 ± 2.17 days, *p* < 0.001) and lower complication rate (14.1% vs. 31.4%, *p* = 0.006). Gastrointestinal function recovery was accelerated, with earlier time to first flatus (24.95 ± 5.16 vs. 36.28 ± 7.16 h, *p* < 0.001) and first ambulation (19.72 ± 4.54 vs. 29.28 ± 5.11 h, *p* < 0.001). Nutritional markers including serum albumin (34.94 ± 4.59 vs. 32.18 ± 4.38 g/L, *p* < 0.001) and prealbumin (205.02 ± 44.62 vs. 178.63 ± 43.31 mg/L, *p* < 0.001) were significantly higher. Psychological outcomes showed improvement with lower anxiety (HAMA: 10.08 ± 4.24 vs. 15.77 ± 4.48, *p* < 0.001) and depression scores (HAMD: 8.87 ± 5.01 vs. 12.45 ± 4.99, *p* < 0.001). Patient satisfaction was significantly higher (91.72 ± 5.73 vs. 80.07 ± 6.22, *p* < 0.001). After multivariable adjustment for age, sex, BMI, TNM stage, surgical factors and comorbidities, the intervention remained independently associated with shorter hospital stay (adjusted *β* = −3.13 days, 95% CI -3.67 to −2.59) and lower odds of complications (adjusted OR 0.25, 95% CI 0.10–0.58); propensity-score-matched and IPTW analyses yielded concordant estimates. Baseline nutritional and psychological values were comparable between groups, and all significant differences persisted after false-discovery-rate correction.

**Conclusion:**

In this historical cohort study, comprehensive ERAS-based nursing intervention was associated with improved postoperative recovery after radical gastrectomy. Because of the non-randomized, single-centre design and the potential for residual and temporal confounding, these findings should be regarded as hypothesis-generating, and prospective multicentre randomized trials are needed to confirm effectiveness before widespread implementation.

## Introduction

Gastric cancer represents one of the most prevalent malignancies worldwide, with particularly high incidence rates observed in East Asian countries including China, Japan, and South Korea (1, 2). Despite significant advances in diagnostic techniques and therapeutic strategies, gastric cancer continues to rank among the leading causes of cancer-related mortality globally, accounting for approximately 769,000 deaths annually (3). Surgical resection remains the cornerstone of curative treatment for patients with resectable gastric cancer, with radical gastrectomy combined with systematic lymphadenectomy representing the standard surgical approach (4, 5).

However, radical gastrectomy is associated with substantial physiological stress and considerable risk of postoperative complications. Patients commonly experience a spectrum of challenges during the recovery period, including delayed gastrointestinal function recovery, nutritional deterioration, wound-related complications, and psychological distress (6–8). Previous studies have reported postoperative complication rates ranging from 15 to 35% following radical gastrectomy, with pulmonary infections, anastomotic complications, and wound infections being the most frequently encountered adverse events (9, 10).

The concept of enhanced recovery after surgery (ERAS), initially introduced by Kehlet in colorectal surgery, has revolutionized perioperative care paradigms across multiple surgical specialties (11, 12). ERAS protocols encompass a multimodal, evidence-based approach that integrates preoperative optimization, intraoperative management strategies, and postoperative rehabilitation measures aimed at reducing surgical stress, accelerating functional recovery, and minimizing complications (13, 14). The implementation of ERAS principles in gastric cancer surgery has gained increasing attention, with emerging evidence suggesting favorable outcomes in terms of reduced hospital stay, lower complication rates, and improved patient satisfaction (15–17).

Nursing care constitutes an integral and indispensable component of ERAS implementation. The nursing staff plays a pivotal role in patient education, symptom monitoring, early mobilization facilitation, nutritional support, and psychological counseling throughout the perioperative period (18, 19). Comprehensive nursing intervention programs that systematically incorporate ERAS principles have demonstrated promising results in enhancing postoperative recovery across various surgical populations. Nevertheless, evidence specifically addressing the effectiveness of such nursing interventions in Chinese gastric cancer patients remains relatively limited (20, 21).

Importantly, much of the existing evidence derives from general ERAS protocols rather than from specifically nursing-led interventions, and rigorous evaluation of nurse-delivered ERAS care in gastric cancer patients, with adjustment for confounding, remains scarce, despite established ERAS Society recommendations for gastrectomy (22).

The present study was designed to evaluate the effectiveness of a comprehensive nursing intervention program based on ERAS principles in patients undergoing radical gastrectomy for gastric cancer. We hypothesized that patients receiving the comprehensive nursing intervention would demonstrate superior postoperative outcomes compared to those receiving conventional nursing care, as reflected by shortened hospital stay, reduced complications, accelerated gastrointestinal function recovery, improved nutritional status, enhanced psychological well-being, and greater patient satisfaction.

## Methods

### Study design and setting

This retrospective historical cohort study was conducted at the Department of Gastrointestinal Surgery, The First People’s Hospital of Jiangxia District, a tertiary academic medical center, between January 2022 and December 2024. The study protocol was approved by the Institutional Review Board (Approval No.: 2025 K019), and the requirement for individual informed consent was waived due to the retrospective nature of the study. All procedures were performed in accordance with the Declaration of Helsinki.

The study was approved on 15 April 2025 (Approval No.: 2025 K019). This study is reported in accordance with the STROBE statement for observational studies (EQUATOR network) (23), and the completed checklist is provided as Supplementary Table S2.

Because the control group was treated in 2022 and the intervention group in 2023–2024, this is a historical (before-after) cohort study rather than a contemporaneous comparison. This design is susceptible to temporal confounding, as secular changes in surgical technique, anesthetic and perioperative management, complication surveillance, staff experience, and discharge practice may have occurred between periods; we therefore describe the design as a historical cohort study, adjusted for measured confounders in the analyses below, and temper causal interpretation throughout.

### Study population

Patients were eligible for inclusion if they met the following criteria: (1) histologically confirmed diagnosis of gastric adenocarcinoma; (2) underwent elective radical gastrectomy with D2 lymphadenectomy; (3) age between 18 and 80 years; (4) American Society of Anesthesiologists physical status classification I-III; and (5) complete medical records available for data extraction. Exclusion criteria included: (1) emergency surgery; (2) presence of distant metastasis; (3) concurrent malignancies; (4) severe cardiopulmonary dysfunction; (5) preoperative psychiatric disorders requiring pharmacotherapy; and (6) incomplete follow-up data.

Based on the nursing care protocol implemented during hospitalization, patients were categorized into two groups: the control group comprised patients who received conventional perioperative nursing care from January 2022 to December 2022, while the intervention group included patients who received the comprehensive ERAS-based nursing intervention program from January 2023 to December 2024. Patients who had received neoadjuvant chemotherapy or radiotherapy were not included. Throughout the study period, operations were performed by the same core team of gastrointestinal surgeons and anesthetists, and perioperative care was delivered by the same nursing department, minimizing provider-level differences between groups.

Discharge criteria were standardized and required adequate oral intake without intravenous support, satisfactory pain control on oral analgesia, passage of flatus or stool, independent mobilization, absence of fever or an untreated complication, and patient agreement. Postoperative hospital stay was defined as the number of days from the day of surgery to the day of discharge.

## Nursing intervention protocols

### Conventional nursing care (control group)

Patients received standard perioperative nursing care according to institutional protocols, including routine admission assessment, patient education regarding surgical procedures, conventional bowel preparation, standard monitoring of vital signs, wound care, traditional analgesic protocols, and gradual progression of oral intake based on the surgeon’s discretion. Ambulation was typically encouraged starting from postoperative day 2–3.

### Comprehensive ERAS-based nursing intervention (intervention group)

The comprehensive nursing intervention program consisted of three phases (24, 25). (1) Preoperative Phase: structured education sessions covering disease knowledge and perioperative expectations, individualized nutritional assessment with oral nutritional supplements when indicated, psychological preparation including anxiety assessment using HAMA and supportive counseling, and instruction on early mobilization techniques and breathing exercises. (2) Intraoperative Coordination: seamless communication with surgical and anesthesia teams regarding patient-specific concerns, temperature monitoring and maintenance protocols. (3) Postoperative Phase: multimodal analgesia incorporating patient-controlled analgesia and complementary approaches including positioning guidance and relaxation techniques, early mobilization initiated within 6 h postoperatively with progressive ambulation goals, early oral feeding protocols beginning with clear liquids on the first postoperative day and advancing as tolerated, continuous nutritional monitoring with oral nutritional supplements, and psychological support through daily nursing rounds and family involvement (26, 27).

The full protocol, including each component, its timing, the responsible staff, implementation criteria, and intended adherence targets, is detailed in Supplementary Table S1. Because the intervention was reconstructed retrospectively from the electronic record, prospective per-component adherence logs (e.g., proportion mobilized within a target interval, oral intake achieved on postoperative day 1, and receipt of multimodal analgesia and oral nutritional supplements) were not systematically maintained; the absence of protocol-adherence data is acknowledged as an important limitation.

### Data collection and outcome measures

Demographic and clinical data were extracted from electronic medical records by trained research nurses. The primary outcomes were postoperative hospital stay (defined as days from surgery to discharge) and the incidence of postoperative complications (including pulmonary infection, anastomotic leakage, bleeding, wound infection, and intestinal obstruction). Secondary outcomes encompassed: (1) gastrointestinal function recovery parameters, including time to first flatus, first defecation, first ambulation, and resumption of oral intake; (2) nutritional status indicators assessed on postoperative day 7, including serum albumin (ALB), prealbumin (PA), transferrin (TRF), and total lymphocyte count (TLC); (3) psychological status evaluated at discharge using the Hamilton Anxiety Rating Scale (HAMA) and Hamilton Depression Rating Scale (HAMD) (26); (4) postoperative pain assessed using the Visual Analog Scale (VAS, 0–10) on postoperative days 1, 2, and 3; and (5) patient satisfaction score using a standardized questionnaire (scale 0–100).

Postoperative complications were ascertained during the index hospitalization and within 30 days of surgery. Pulmonary infection was defined by new respiratory symptoms with a radiographic infiltrate and initiation of antibiotics; anastomotic leakage by clinical, radiological, or operative confirmation; postoperative bleeding by need for transfusion, intervention, or reoperation; wound infection by purulent discharge or the need to open the wound; and intestinal obstruction and delayed gastric emptying by combined clinical and radiological criteria. Complications were defined using the Clavien-Dindo framework; however, because data were extracted retrospectively, formal Clavien-Dindo grades could not be reliably reconstructed for every event and are therefore not reported as a graded distribution (see Limitations). Nutritional status was assessed with routinely available laboratory parameters (serum albumin, prealbumin, transferrin, and total lymphocyte count) measured both preoperatively and on postoperative day 7, enabling analysis of change from baseline. Morphofunctional assessment (bioelectrical impedance analysis, muscle ultrasound, or dynamometry), validated nutritional-risk scales such as the GLIM criteria, and serum total cholesterol and triglycerides were not part of routine documentation during the study period. Patient satisfaction was assessed at discharge using an institutional 0–100 perioperative nursing-satisfaction questionnaire covering communication, responsiveness, education, comfort, and overall care; this instrument has not been formally validated with published reliability and validity metrics, which is acknowledged as a limitation.

### Statistical analysis

Statistical analyses were performed using Python 3.11 (SciPy and statsmodels). Continuous variables are presented as mean±SD and compared with the independent-samples *t*-test; categorical variables are presented as *n* (%) and compared with the chi-square or Fisher’s exact test. For all outcomes, effect sizes (Cohen’s d for continuous outcomes; odds ratio or mean difference for binary and continuous outcomes, respectively) are reported with 95% confidence intervals in addition to *p* values. Postoperative hospital stay and complication rate were pre-specified as primary outcomes; all other outcomes were secondary and interpreted as hypothesis-generating. To address multiplicity among secondary outcomes, *p* values were adjusted using the Benjamini-Hochberg false-discovery-rate procedure. To mitigate confounding inherent to the non-randomized historical design, multivariable linear (continuous outcomes) and logistic (complications) regression models were fitted, adjusting for age, sex, BMI, TNM stage, surgery type, surgical approach, ASA class, operation time, blood loss, hypertension, and diabetes. As sensitivity analyses, a propensity score for group assignment was estimated from the same covariates, and (i) 1:1 nearest-neighbor matching within a caliper of 0.2 SD of the logit propensity score and (ii) stabilized inverse-probability-of-treatment weighting were performed. Repeated VAS pain scores on postoperative days 1–3 were analyzed with a linear mixed-effects model with fixed effects for group, time, and their interaction and a random intercept per patient. All tests were two-sided, and *p* < 0.05 was considered statistically significant.

Sample-size considerations. As a retrospective study, no *a priori* sample-size calculation was performed. A post-hoc analysis indicated that the available sample (86 vs. 92) provided greater than 99% power to detect the observed difference in hospital stay and approximately 80% power to detect the observed difference in complication rate (31.4% vs. 14.1%) at a two-sided alpha of 0.05, and was powered to detect a standardized between-group difference of Cohen’s d ≈ 0.42 for continuous secondary outcomes. Smaller effects, such as the transferrin difference, may be underpowered and are interpreted cautiously.

## Results

### Baseline characteristics

A total of 178 patients were analyzed (control *n* = 86, intervention *n* = 92; Table 1). Age (62.03 ± 9.12 vs. 62.10 ± 8.64 years, *p* = 0.962), BMI (*p* = 0.994), TNM stage (*p* = 0.980), surgery type (*p* = 0.876), surgical approach (laparoscopic 68.6% vs. 70.7%, *p* = 0.767), ASA class (*p* = 0.279), hypertension (*p* = 0.832), diabetes (*p* = 0.345), operation time (*p* = 0.306), and blood loss (*p* = 0.058) were comparable between groups. Importantly, preoperative nutritional (albumin 38.67 ± 3.86 vs. 38.93 ± 4.06 g/L, *p* = 0.655; prealbumin, transferrin, and TLC all *p* > 0.10) and psychological indices (HAMA 18.41 ± 4.63 vs. 17.39 ± 4.27, *p* = 0.129; HAMD *p* = 0.826) did not differ between groups, indicating comparable baseline status. The groups differed in sex distribution, with more men in the control group (62.8% vs. 46.7%, *p* = 0.032); this imbalance was addressed in the adjusted analyses described below.

**Table 1:** Baseline characteristics of the study population.

Characteristic	Control (*n* = 86)	Intervention (*n* = 92)	*t*/*χ*^2^	*p* value
Age (years)	62.03 ± 9.12	62.10 ± 8.64	0.047	0.962
Male, n (%)	54 (62.8)	43 (46.7)	4.619	0.032
BMI (kg/m^2^)	23.06 ± 3.07	23.06 ± 3.13	0.007	0.994
TNM Stage, *n* (%)			0.040	0.980
Stage I	22 (25.6)	23 (25.0)		
Stage II	39 (45.3)	41 (44.6)		
Stage III	25 (29.1)	28 (30.4)		
Surgery type, n (%)			0.264	0.876
Distal gastrectomy	45 (52.3)	51 (55.4)		
Total gastrectomy	35 (40.7)	34 (37.0)		
Proximal gastrectomy	6 (7.0)	7 (7.6)		
Operation time (min)	220.30 ± 46.92	213.22 ± 45.10	1.027	0.306
Blood loss (mL)	116.06 ± 48.40	130.10 ± 49.56	1.902	0.058
Surgical approach, *n* (%)			0.088	0.767
Laparoscopic	59 (68.6)	65 (70.7)		
Open	27 (31.4)	27 (29.3)		
ASA class, *n* (%)			2.550	0.279
I	14 (16.3)	24 (26.1)		
II	59 (68.6)	56 (60.9)		
III	13 (15.1)	12 (13.0)		
Hypertension, *n* (%)	24 (27.9)	27 (29.3)	0.045	0.832
Diabetes, *n* (%)	9 (10.5)	14 (15.2)	0.892	0.345
Preoperative albumin (g/L)	38.67 ± 3.86	38.93 ± 4.06	0.447	0.655
Preoperative prealbumin (mg/L)	245.05 ± 40.95	242.85 ± 41.08	0.357	0.721
Preoperative transferrin (g/L)	2.36 ± 0.38	2.31 ± 0.39	0.860	0.391
Preoperative TLC (×10^9^/L)	1.63 ± 0.50	1.75 ± 0.47	1.646	0.102
Preoperative HAMA score	18.41 ± 4.63	17.39 ± 4.27	1.524	0.129
Preoperative HAMD score	15.26 ± 4.50	15.40 ± 4.34	0.221	0.826

Continuous variables are presented as mean±SD; categorical variables as n (%). BMI, body mass index; TNM, tumor-node-metastasis. ASA, American Society of Anesthesiologists; TLC, total lymphocyte count; HAMA, Hamilton Anxiety Rating Scale; HAMD, Hamilton Depression Rating Scale. *p* values by *t*-test (continuous) or chi-square test (categorical).

### Primary outcomes

**Figure 1:**
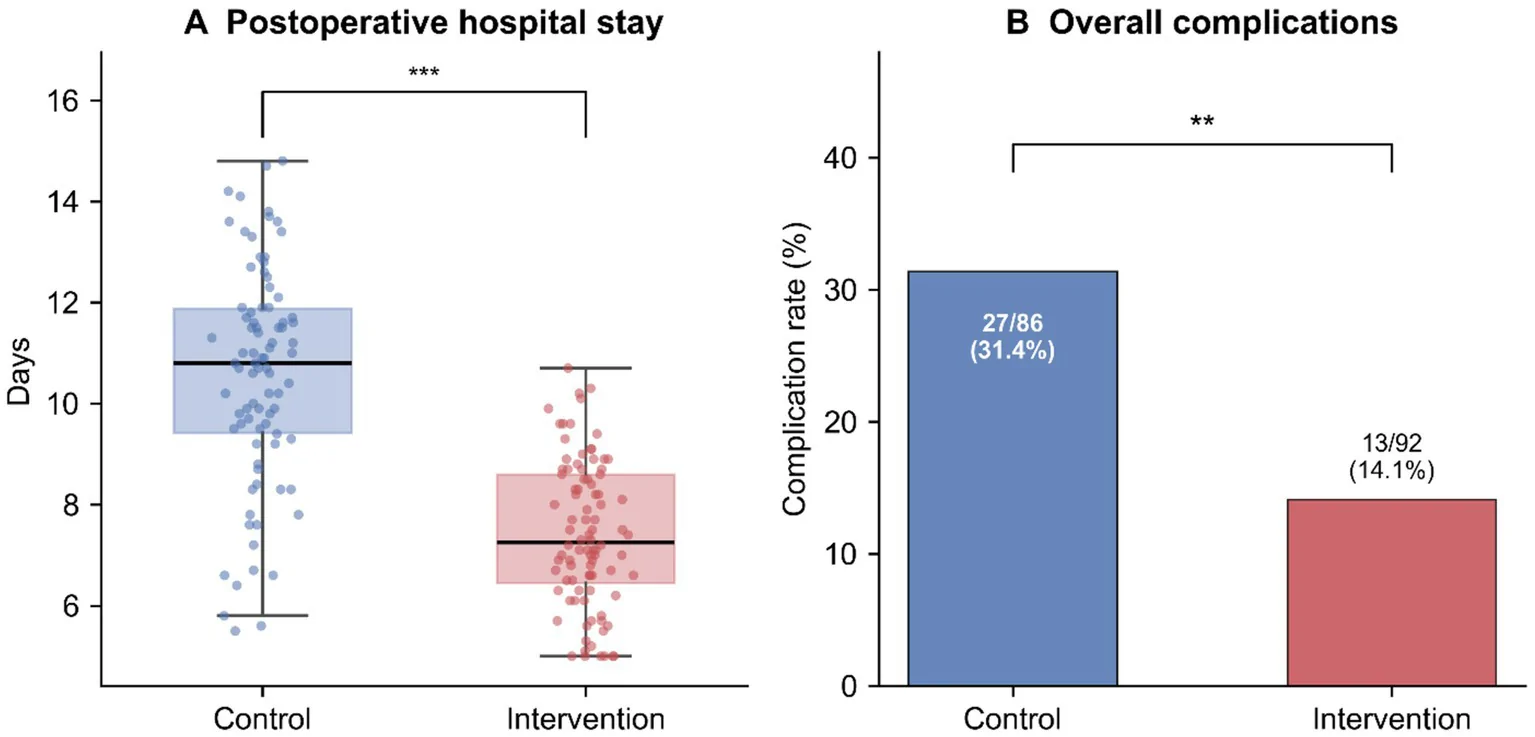
Primary outcomes comparing control and intervention groups. **(A)** Postoperative hospital stay (days); boxes show median and interquartile range with individual patients overlaid as dots, showing a shorter stay in the intervention group (7.42 ± 1.44 vs. 10.56 ± 2.17 days, *p* < 0.001). **(B)** Overall complication rate (%), with event counts shown (14.1% vs. 31.4%, *p* = 0.006). Blue, control; red, intervention. ***p < 0.001, **p < 0.01.

The intervention group had a shorter postoperative hospital stay than the control group (7.42 ± 1.44 vs. 10.56 ± 2.17 days; mean difference −3.14 days, 95% CI -3.69 to −2.60; Cohen’s d = 1.72; *p* < 0.001; Figure 1A). The overall complication rate was lower in the intervention group (13/92, 14.1% vs. 27/86, 31.4%; χ^2^ = 7.61, *p* = 0.006; Figure 1B). By type (recomputed from the source records), the control group had more pulmonary infections [9 (10.5%) vs. 1 (1.1%)], anastomotic leakage [5 (5.8%) vs. 2 (2.2%)], bleeding [6 (7.0%) vs. 2 (2.2%)], and intestinal obstruction [3 (3.5%) vs. 2 (2.2%)]; wound infection was slightly more frequent in the intervention group [5 (5.4%) vs. 3 (3.5%)], and delayed gastric emptying was rare in both (1 each). After multivariable adjustment, the intervention remained independently associated with shorter hospital stay (adjusted *β* = −3.13 days, 95% CI -3.67 to −2.59, *p* < 0.001) and lower odds of complications (adjusted OR 0.25, 95% CI 0.10–0.58, *p* = 0.001). These estimates were confirmed by 1:1 propensity-score matching (67 matched pairs: 7.54 ± 1.37 vs. 10.50 ± 2.16 days, *p* < 0.001; complications 11.9% vs. 32.8%, *p* = 0.004) and by IPTW (hospital stay *β* = −3.12, *p* < 0.001; complication OR 0.31, 95% CI 0.15–0.66, *p* = 0.003; Table 2). Detailed data are presented in Table 3.

**Table 2:** Covariate-adjusted and propensity-score sensitivity analyses for key outcomes.

Outcome	Unadjusted effect (95% CI)	Adjusted effect (95% CI)	Adjusted *P*
Hospital stay (days)	−3.14 (−3.69, −2.60)	−3.13 (−3.67, −2.59)	<0.001
Complications (OR)	0.36 (0.17, 0.76)	0.25 (0.10, 0.58)	0.001
First flatus (hours)	−11.33 (−13.19, −9.47)	−10.92 (−12.84, −9.01)	<0.001
Albumin, day 7 (g/L)	2.77 (1.44, 4.09)	2.69 (1.29, 4.08)	<0.001
HAMA score	−5.69 (−6.98, −4.40)	−5.72 (−7.07, −4.38)	<0.001
Satisfaction score	11.65 (9.88, 13.42)	11.69 (9.87, 13.52)	<0.001

Effects are mean differences (intervention minus control) for continuous outcomes and odds ratios (OR) for complications. Adjusted models included age, sex, BMI, TNM stage, surgery type, surgical approach, ASA class, operation time, blood loss, hypertension, and diabetes. Concordant estimates were obtained from 1:1 propensity-score matching (67 pairs) and inverse-probability-of-treatment weighting (hospital stay *β* = −3.12; complication OR 0.31, 95% CI 0.15–0.66).

**Table 3:** Primary outcomes and gastrointestinal function recovery.

Variable	Control (*n* = 86)	Intervention (*n* = 92)	*t*/χ^2^	*p* value
Primary outcomes				
Hospital stay (days)	10.56 ± 2.17	7.42 ± 1.44	11.475	<0.001
Complications, *n* (%)	27 (31.4)	13 (14.1)	7.610*	0.006
Pulmonary infection	9 (10.5)	1 (1.1)		
Anastomotic leakage	5 (5.8)	2 (2.2)		
Bleeding	6 (7.0)	2 (2.2)		
Wound infection	3 (3.5)	5 (5.4)		
Intestinal obstruction	3 (3.5)	2 (2.2)		
Delayed gastric emptying	1 (1.2)	1 (1.1)		
GI function recovery				
First flatus (hours)	36.28 ± 7.16	24.95 ± 5.16	12.166	<0.001
First defecation (hours)	64.87 ± 11.85	49.01 ± 10.34	9.529	<0.001
First ambulation (hours)	29.28 ± 5.11	19.72 ± 4.54	13.196	<0.001
Oral intake (days)	4.66 ± 1.11	3.19 ± 0.93	9.576	<0.001

*χ^2^ value for categorical variable. Continuous variables are presented as mean±SD. GI, gastrointestinal.

### Gastrointestinal function recovery

**Figure 2:**
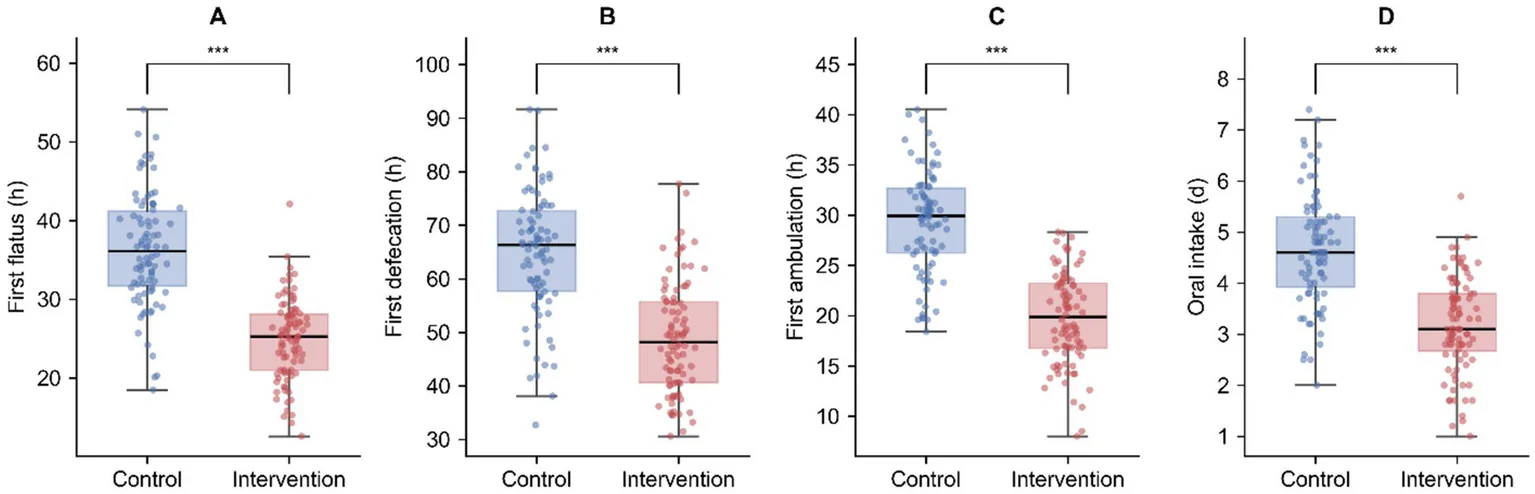
Gastrointestinal function recovery parameters. **(A)** Time to first flatus (hours); **(B)** Time to first defecation (hours); **(C)** Time to first ambulation (hours); **(D)** Time to oral intake (days). All parameters recovered faster in the intervention group (all *p* < 0.001). Boxes show median and interquartile range; blue, control; red, intervention. ***p < 0.001.

The intervention group exhibited significantly accelerated gastrointestinal function recovery across all measured parameters (Table 3; Figure 2). Time to first flatus was substantially shorter in the intervention group compared to the control group (24.95 ± 5.16 vs. 36.28 ± 7.16 h, *t* = 12.166, *p* < 0.001), representing a 31.2% reduction. Time to first defecation was significantly reduced (49.01 ± 10.34 vs. 64.87 ± 11.85 h, *t* = 9.529, *p* < 0.001). Early ambulation was achieved significantly earlier in the intervention group (19.72 ± 4.54 vs. 29.28 ± 5.11 h, *t* = 13.196, *p* < 0.001), reflecting the emphasis on early mobilization protocols. Time to resumption of oral intake was also significantly shorter (3.19 ± 0.93 vs. 4.66 ± 1.11 days, *t* = 9.576, *p* < 0.001). All four differences remained significant after Benjamini-Hochberg correction and after multivariable adjustment (all adjusted *p* < 0.001; Table 2).

### Nutritional status

**Figure 3:**
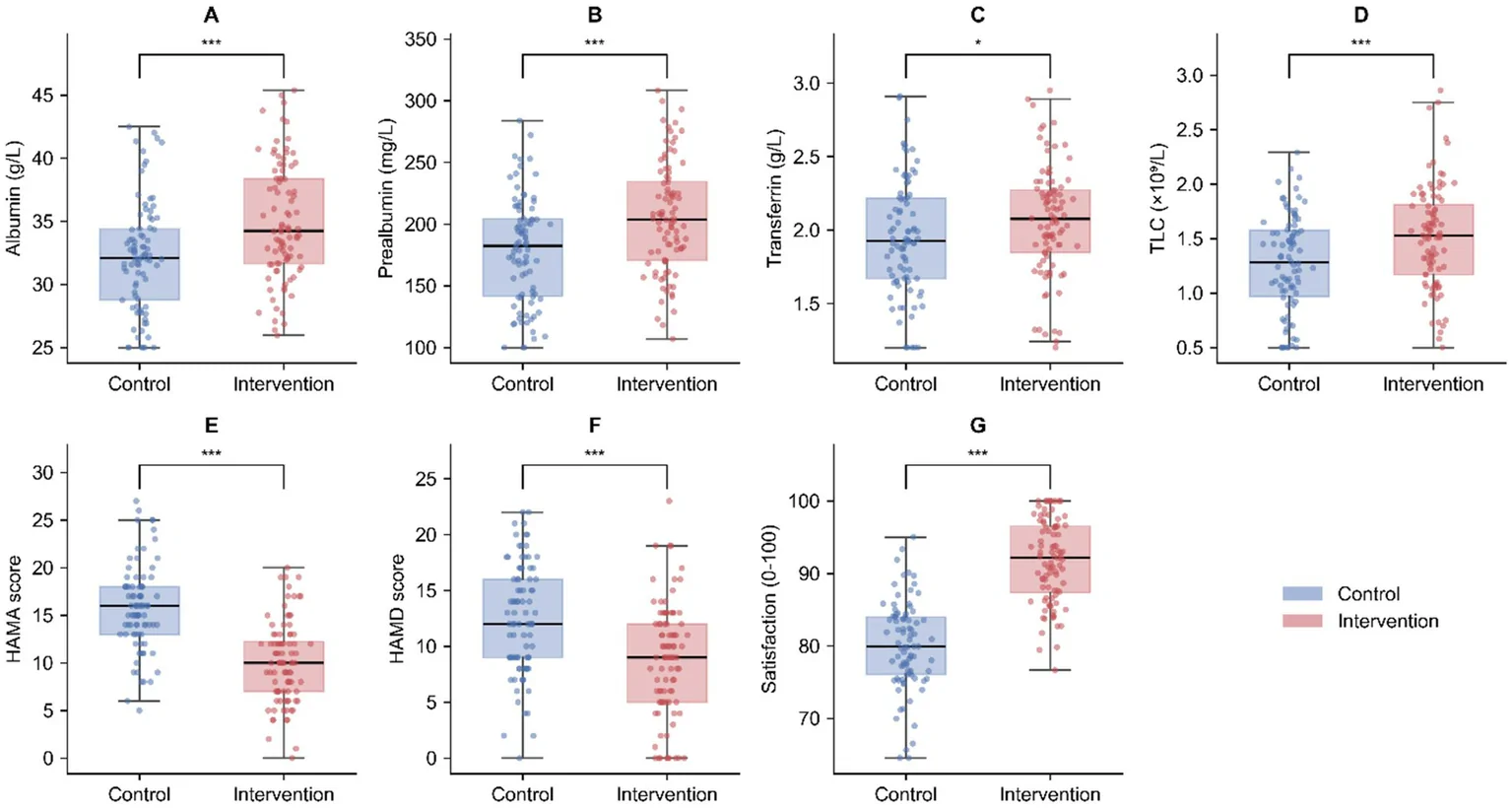
Nutritional and psychological outcomes, shown as separate panels with independent axes; each dot is one patient. **(A–D)** Nutritional markers on postoperative day 7: albumin (*p* < 0.001), prealbumin (*p* < 0.001), transferrin (*p* = 0.049), and total lymphocyte count (*p* < 0.001). **(E–G)** Psychological scores: HAMA (*p* < 0.001), HAMD (*p* < 0.001), and satisfaction (*p* < 0.001). Boxes show median and interquartile range; whiskers show the 1.5 × IQR range; overlaid points show individual patients (blue, control; red, intervention). ***p < 0.001, *p < 0.05.

Postoperative nutritional indicators assessed on day 7 showed significant differences between groups, with the intervention group demonstrating superior nutritional status (Table 4; Figure 3A). Serum albumin levels were significantly higher in the intervention group (34.94 ± 4.59 vs. 32.18 ± 4.38 g/L, *t* = 4.109, *p* < 0.001). Similarly, prealbumin levels were elevated in the intervention group (205.02 ± 44.62 vs. 178.63 ± 43.31 mg/L, *t* = 3.999, *p* < 0.001). Transferrin levels showed improvement (2.06 ± 0.38 vs. 1.95 ± 0.39 g/L, *t* = 1.986, *p* = 0.049), and total lymphocyte count was significantly higher (1.52 ± 0.48 vs. 1.27 ± 0.45 × 10^9^/L, *t* = 3.564, *p* < 0.001), indicating enhanced immune nutritional status.

**Table 4:** Nutritional status on postoperative day 7.

Variable	Control (*n* = 86)	Intervention (*n* = 92)	*t*	*p* value
Albumin (g/L)	32.18 ± 4.38	34.94 ± 4.59	4.109	<0.001
Prealbumin (mg/L)	178.63 ± 43.31	205.02 ± 44.62	3.999	<0.001
Transferrin (g/L)	1.95 ± 0.39	2.06 ± 0.38	1.986	0.049
TLC (×10^9^/L)	1.27 ± 0.45	1.52 ± 0.48	3.564	<0.001

TLC, total lymphocyte count. Values are presented as mean ± SD.

**Figure 4:**
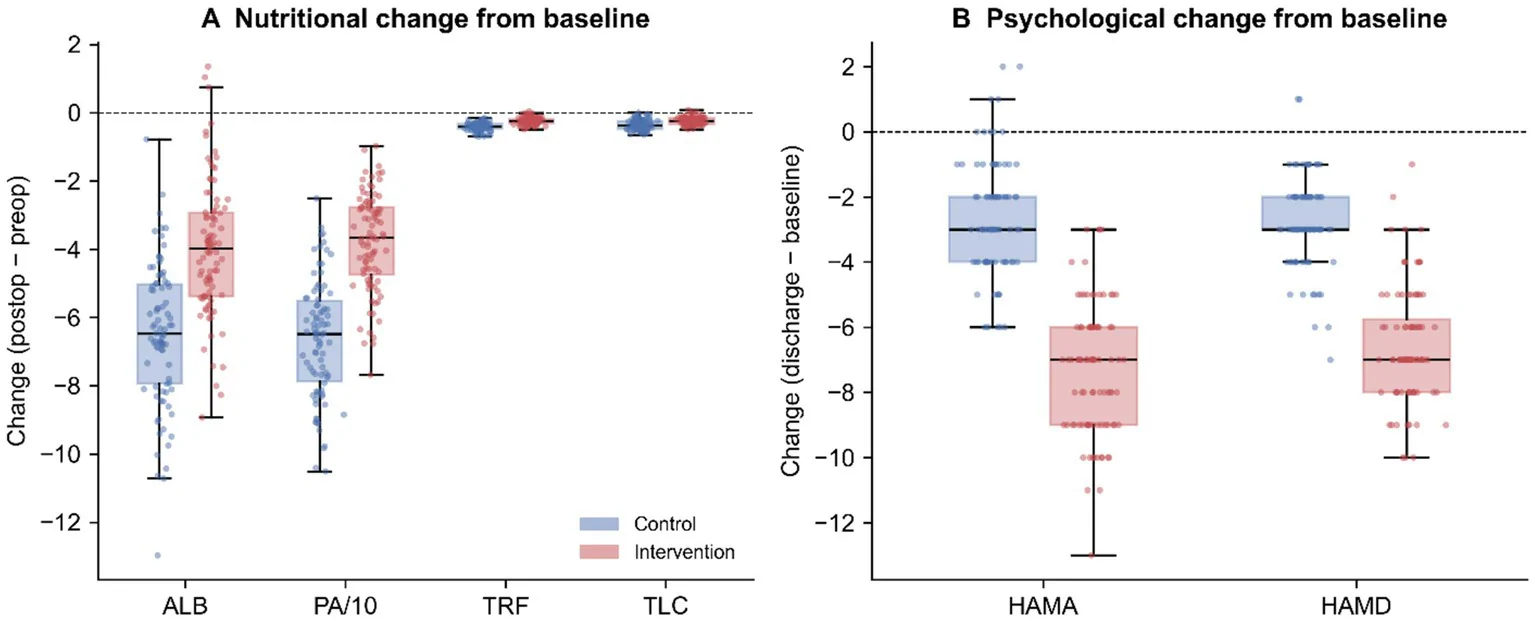
Change from baseline in nutritional and psychological parameters, plotted as individual patient changes. **(A)** Change (postoperative day 7 minus preoperative) in nutritional markers (prealbumin shown as change/10 for display). **(B)** Change from baseline to discharge in HAMA and HAMD scores. Boxes show median and interquartile range; each dot is one patient (blue, control; red, intervention). The intervention group showed significantly smaller nutritional declines and greater reductions in anxiety and depression (all *p* < 0.001). Baseline values were comparable (Table 5).

Preoperative values were comparable between groups (Table 5). Analyzed as change from baseline, the intervention group showed significantly smaller declines in albumin (*Δ* − 3.99 ± 1.96 vs. − 6.49 ± 2.07 g/L, *p* < 0.001), prealbumin (Δ -37.84 ± 14.16 vs. − 66.42 ± 17.31 mg/L, *p* < 0.001), transferrin, and total lymphocyte count (both *p* < 0.001; Figure 4A), indicating attenuated postoperative nutritional deterioration rather than pre-existing baseline differences. All differences persisted after false-discovery-rate correction and multivariable adjustment (Table 2).

**Table 5:** Baseline and change-from-baseline nutritional and psychological parameters.

Parameter	Control baseline	Intervention baseline	*p* (baseline)	Control change	Intervention change	*p* (change)
Albumin (g/L)	38.67 ± 3.86	38.93 ± 4.06	0.655	−6.49 ± 2.07	−3.99 ± 1.96	<0.001
Prealbumin (mg/L)	245.05 ± 40.95	242.85 ± 41.08	0.721	−66.42 ± 17.31	−37.84 ± 14.16	<0.001
Transferrin (g/L)	2.36 ± 0.38	2.31 ± 0.39	0.391	−0.41 ± 0.12	−0.24 ± 0.11	<0.001
TLC (×10^9^/L)	1.63 ± 0.50	1.75 ± 0.47	0.102	−0.36 ± 0.15	−0.24 ± 0.11	<0.001
HAMA score	18.41 ± 4.63	17.39 ± 4.27	0.129	−2.64 ± 1.76	−7.32 ± 2.00	<0.001
HAMD score	15.26 ± 4.50	15.40 ± 4.34	0.826	−2.80 ± 1.38	−6.53 ± 1.75	<0.001

Change = postoperative (day 7 for nutritional markers; discharge for psychological scores) minus preoperative value. Values are mean±SD. Baseline values did not differ between groups, indicating comparable pre-intervention status. TLC, total lymphocyte count; HAMA, Hamilton Anxiety Rating Scale; HAMD, Hamilton Depression Rating Scale.

### Psychological outcomes

Psychological assessment at discharge revealed significantly better outcomes in the intervention group (Table 6; Figure 3B). HAMA scores were substantially lower in the intervention group (10.08 ± 4.24 vs. 15.77 ± 4.48, *t* = 8.711, *p* < 0.001), indicating reduced anxiety levels. HAMD scores were also significantly lower (8.87 ± 5.01 vs. 12.45 ± 4.99, *t* = 4.780, *p* < 0.001), reflecting improved depressive symptoms. Baseline HAMA and HAMD scores were comparable between groups (Table 5); from baseline to discharge, anxiety and depression decreased more in the intervention group (ΔHAMA −7.32 ± 2.00 vs. − 2.64 ± 1.76; ΔHAMD -6.53 ± 1.75 vs. − 2.80 ± 1.38; both *p* < 0.001; Figure 4B), and both associations persisted after multivariable adjustment (Table 2).

**Table 6:** Psychological outcomes at discharge.

Variable	Control (*n* = 86)	Intervention (*n* = 92)	*t*	*p* value
HAMA score	15.77 ± 4.48	10.08 ± 4.24	8.711	<0.001
HAMD score	12.45 ± 4.99	8.87 ± 5.01	4.780	<0.001
Satisfaction score	80.07 ± 6.22	91.72 ± 5.73	13.005	<0.001

HAMA, Hamilton Anxiety Rating Scale (higher scores indicate greater anxiety); HAMD, Hamilton Depression Rating Scale (higher scores indicate greater depression); Satisfaction score ranges from 0 to 100 (higher scores indicate greater satisfaction). Values are presented as mean±SD.

### Pain scores and patient satisfaction

**Figure 5:**
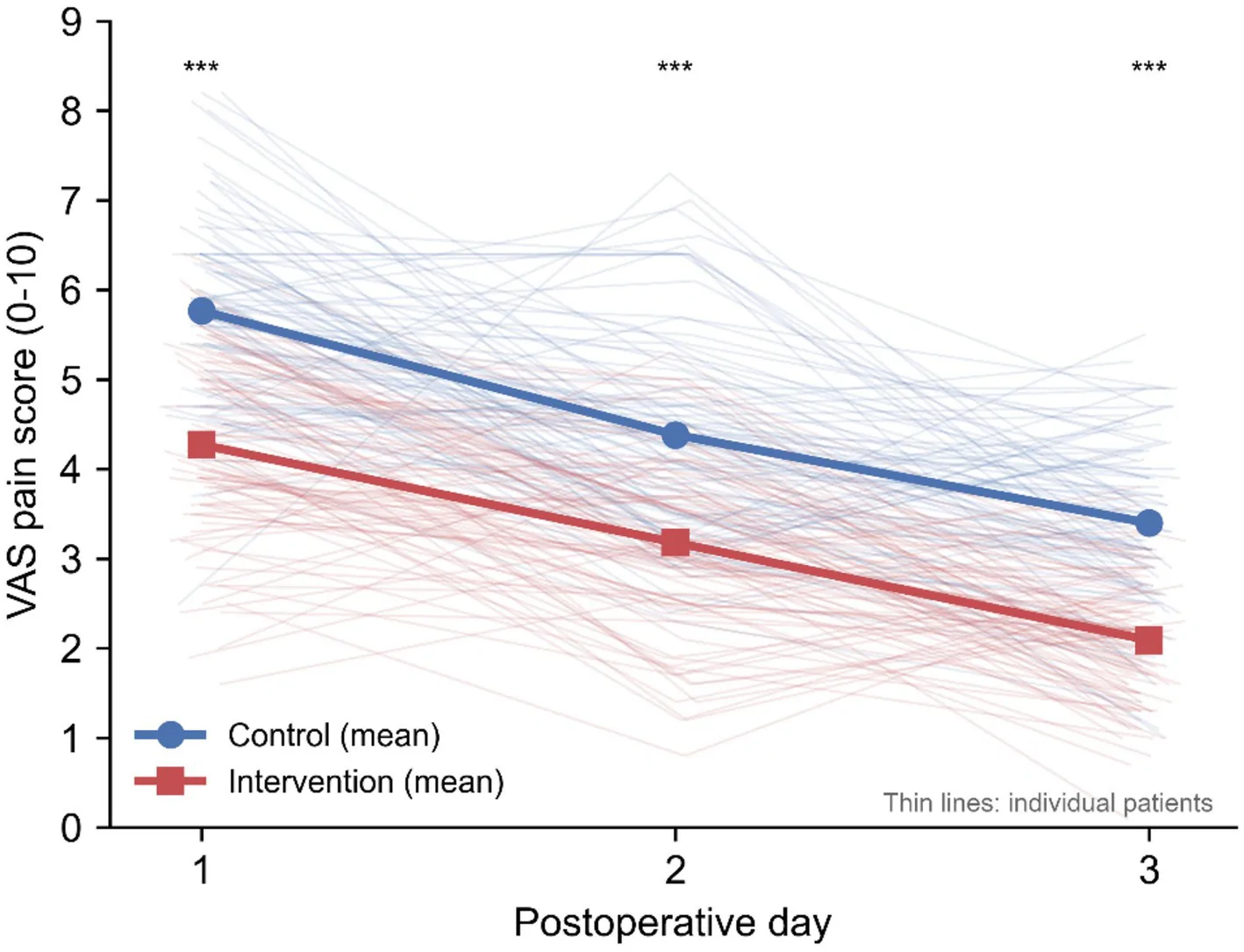
Postoperative pain trajectory assessed by Visual Analog Scale (VAS, 0–10) on postoperative days 1–3. Thin lines show individual patient trajectories; thick lines show group means (blue, control; red, intervention). The intervention group had consistently lower pain (all *p* < 0.001; mixed-effects group effect *p* < 0.001, group-by-time interaction *p* = 0.38).

VAS pain scores were consistently lower in the intervention group across all assessment time points (Table 7; Figure 5): postoperative day 1 (4.27 ± 1.06 vs. 5.77 ± 1.06, *t* = 9.412, *p* < 0.001), day 2 (3.18 ± 0.98 vs. 4.38 ± 1.12, t = 7.627, *p* < 0.001), and day 3 (2.09 ± 0.73 vs. 3.40 ± 0.96, *t* = 10.300, *p* < 0.001). The intervention group demonstrated a more rapid decline in pain scores over time. Patient satisfaction scores were significantly higher in the intervention group (91.72 ± 5.73 vs. 80.07 ± 6.22, *t* = 13.005, *p* < 0.001), reflecting comprehensive improvements in the overall care experience.

**Table 7:** Postoperative pain scores (visual analog scale).

Time Point	Control (*n* = 86)	Intervention (*n* = 92)	*t*	*p* value
Postoperative day 1	5.77 ± 1.06	4.27 ± 1.06	9.412	<0.001
Postoperative day 2	4.38 ± 1.12	3.18 ± 0.98	7.627	<0.001
Postoperative day 3	3.40 ± 0.96	2.09 ± 0.73	10.300	<0.001

VAS scores range from 0 (no pain) to 10 (worst imaginable pain). Values are presented as mean±SD.

A linear mixed-effects model confirmed a significant overall group effect (−1.52 points, 95% CI -1.97 to −1.08, *p* < 0.001) and a significant decline over time (*p* < 0.001); the group-by-time interaction was not significant (*p* = 0.38), indicating consistently lower pain scores in the intervention group with parallel resolution across the three postoperative days.

## Discussion

The present study demonstrates that comprehensive nursing intervention based on ERAS principles was associated with improved postoperative outcomes in patients undergoing radical gastrectomy for gastric cancer. Our findings reveal substantial benefits across multiple domains, including shortened hospital stay, reduced complication rates, accelerated gastrointestinal function recovery, improved nutritional status, enhanced psychological well-being, and increased patient satisfaction. Notably, the association with improved primary outcomes persisted across multivariable regression, propensity-score matching, and inverse-probability weighting, which strengthens internal validity; nevertheless, given the observational, historical design, residual and unmeasured confounding cannot be excluded and causal inference is not warranted.

The observed reduction in postoperative hospital stay (7.42 vs. 10.56 days, representing a 29.7% decrease) is consistent with previous studies implementing ERAS protocols in gastric cancer surgery. Song et al. (15) reported similar findings with hospital stay of 7.2 ± 1.5 days in the ERAS group versus 10.5 ± 2.1 days in the conventional care group. Wu et al. (16) demonstrated comparable results with hospital stay reduced from 12.50 to 8.75 days following ERAS nursing combined with early enteral nutrition. The shortened hospital stay can be attributed to the multimodal approach of comprehensive nursing intervention, including early mobilization, optimized pain management, and accelerated nutritional rehabilitation, which collectively facilitate faster physiological recovery (28, 29).

The significant reduction in complication rates (14.1% vs. 31.4%) observed in our study aligns with the findings of recent meta-analyses examining ERAS implementation in gastrointestinal surgery. Li et al. (17) reported that enhanced recovery protocols, including avoiding routine abdominal drainage, led to significantly lower complication rates in gastric cancer patients. The comprehensive nursing intervention likely contributed to reduced complications through enhanced patient monitoring, systematic assessment protocols, early identification of warning signs, and prompt intervention when complications were detected. Particularly noteworthy was the reduction in pulmonary infections, which may be attributed to the emphasis on early mobilization and breathing exercises (30).

The accelerated gastrointestinal function recovery demonstrated in our study supports the effectiveness of early mobilization and early oral feeding protocols. Tang et al. (14) reported that ERAS-based multidisciplinary collaboration resulted in significantly shorter times to first flatus and defecation. Yuan et al. (13) similarly found that ERAS implementation in laparoscopic gastric cancer surgery promoted gastrointestinal function recovery. The earlier time to first ambulation (19.72 vs. 29.28 h) reflects the emphasis on early mobilization within the comprehensive nursing intervention protocol, which has been shown to enhance gastrointestinal motility, reduce postoperative ileus, and promote overall recovery (31).

The improved nutritional status observed in the intervention group, as reflected by higher albumin and prealbumin levels, is particularly noteworthy given the high prevalence of malnutrition among gastric cancer patients. Lu et al. (20) demonstrated that ERAS continuity nursing care significantly improved nutritional markers in elderly gastric cancer patients. Shin (28) emphasized the importance of perioperative nutritional treatment for gastric cancer patients. The comprehensive nursing intervention in our study incorporated systematic nutritional assessment, early oral feeding protocols, and oral nutritional supplements, which collectively contributed to better nutritional outcomes and may reduce the risk of nutrition-related complications.

The psychological benefits demonstrated in our study, with significantly lower anxiety and depression scores at discharge, underscore the importance of addressing psychological well-being in perioperative care. Guo et al. (32) identified that interventions targeting nutrition, anxiety, and family cohesion significantly influenced frailty trajectories in older gastric cancer patients. The psychological support component of our comprehensive nursing intervention, including structured preoperative education, daily nursing rounds with emotional assessment, and family involvement strategies, likely contributed to the observed improvements in psychological outcomes (33).

The substantial improvement in pain management, with consistently lower VAS scores across all postoperative days, reflects the effectiveness of multimodal analgesia incorporated within the comprehensive nursing intervention. In addition to pharmacological approaches including patient-controlled analgesia, the intervention included complementary strategies such as patient education, positioning guidance, and relaxation techniques, which may have enhanced patients’ coping abilities and pain tolerance. These findings align with the growing evidence supporting multimodal pain management approaches in surgical patients.

The significant improvement in patient satisfaction (91.72 vs. 80.07) reflects the cumulative positive impact of enhanced nursing care across multiple domains of the patient experience. This finding underscores the value of comprehensive, patient-centered nursing interventions in improving not only clinical outcomes but also subjective experiences of care quality. Li et al. (21) similarly reported that fast-track rehabilitation combined with humanized nursing improved quality of life in post-surgical gastric cancer patients.

Several caveats temper the interpretation of these findings. The intervention was a multicomponent, ERAS-based care package delivered by a multidisciplinary team, encompassing nutritional support, early mobilization, multimodal analgesia, psychological support, and close coordination with surgeons and anesthetists. It is therefore not possible to isolate the specific contribution of nursing care from the broader institutional ERAS implementation, and the observed benefits should be attributed to the integrated perioperative program rather than to nursing intervention alone. Moreover, because the control and intervention cohorts were treated in consecutive calendar periods (2022 vs. 2023–2024), secular improvements in surgical technique, anesthesia, complication surveillance, and discharge practice may have contributed to the observed differences, a temporal confounding intrinsic to the historical-control design.

Several limitations warrant emphasis. First, this was a retrospective, single-centre study with a historical control group treated in a different calendar period; despite multivariable adjustment, propensity-score matching, and IPTW, residual and temporal confounding cannot be excluded, and causal inference is not warranted. Second, per-component adherence to the ERAS protocol was not prospectively documented, so the degree to which outcomes reflect true protocol compliance is uncertain. Third, the patient-satisfaction questionnaire was an institutional instrument without formally published reliability and validity data. Fourth, nutritional status was assessed only with routine laboratory parameters; morphofunctional assessment (bioimpedance, muscle ultrasound, dynamometry), validated nutritional-risk scales such as the GLIM criteria, and lipid parameters (total cholesterol, triglycerides) were unavailable and should be incorporated into future nursing-led research. Fifth, some clinically relevant complications, including acute urinary retention and unplanned intensive-care-unit admission, were not systematically captured. Sixth, formal Clavien-Dindo grading could not be reliably reconstructed retrospectively. Seventh, long-term outcomes (recurrence, survival, sustained quality of life) were not assessed. Adequately powered, prospective, multicentre randomized trials with prospective adherence monitoring and standardized outcome definitions are needed to confirm these associations.

## Conclusion

In this historical cohort study, comprehensive ERAS-based nursing intervention was associated with shorter postoperative hospital stay, fewer complications, faster gastrointestinal recovery, attenuated nutritional decline, better psychological status, lower pain, and higher satisfaction after radical gastrectomy, and these associations were robust to multivariable and propensity-score adjustment. However, given the retrospective, non-randomized, single-centre design and the potential for residual and temporal confounding, these findings should be considered hypothesis-generating. Prospective, multicentre randomized controlled trials with prospective adherence monitoring are needed to confirm effectiveness before ERAS-based comprehensive nursing protocols can be recommended for widespread implementation as a standard of care.

## Data Availability

The original contributions presented in the study are included in the article/Supplementary material, further inquiries can be directed to the corresponding author.
