# Supplementary material for: The role of comprehensive nursing intervention in postoperative recovery of gastric cancer patients: a retrospective historical cohort study
Source: Front Med (Lausanne). 2026 Aug 11;13:1876528. doi: 10.3389/fmed.2026.1876528 (PMC13503252; doi:10.3389/fmed.2026.1876528)
Supplement: Supplementary file 1 [file Table_1.docx]

# Supplementary Material

*The Role of Comprehensive Nursing Intervention in Postoperative Recovery of Gastric Cancer Patients: A Retrospective Historical Cohort Study*

## Supplementary Table S1. Structure of the comprehensive ERAS-based nursing intervention protocol

| **Component** | **Phase / timing** | **Responsible staff** | **Implementation criteria** | **Intended adherence target** |
| --- | --- | --- | --- | --- |
| Preoperative structured education | Admission to day before surgery | Ward nurse / nurse educator | Standardised session on disease, surgery, recovery expectations, and self-care; teach-back confirmation | All patients |
| Nutritional assessment & oral nutritional supplements (ONS) | Preoperative | Ward nurse / dietitian | Screening of nutritional status; ONS prescribed when intake inadequate or risk identified | All screened; ONS as indicated |
| Psychological preparation | Preoperative | Ward nurse | HAMA-based anxiety assessment and supportive counselling; referral if severe | All patients |
| Mobilisation & breathing-exercise instruction | Preoperative | Ward nurse | Instruction and demonstration of early ambulation and incentive breathing exercises | All patients |
| Reduced fasting / carbohydrate guidance | Night before to morning of surgery | Ward nurse / anaesthesia | Shortened clear-fluid fasting per ERAS where clinically appropriate | As clinically appropriate |
| Intraoperative temperature maintenance & team coordination | Intraoperative | OR nurse / anaesthesia | Active warming and structured handover of patient-specific concerns | All patients |
| Multimodal analgesia | Postoperative day 0 onward | Ward nurse / acute-pain service | Patient-controlled analgesia plus non-pharmacological measures (positioning, relaxation); opioid minimisation | All patients |
| Early mobilisation | From postoperative day 0-1 | Ward nurse | Progressive out-of-bed activity initiated early with daily ambulation goals | All patients, target achieved as tolerated |
| Early oral feeding | From postoperative day 1 | Ward nurse / dietitian | Clear liquids on day 1 advancing as tolerated; continued ONS | All patients as tolerated |
| Breathing exercises / pulmonary care | Postoperative | Ward nurse | Scheduled incentive spirometry and coughing to prevent pulmonary complications | All patients |
| Psychological support & family involvement | Daily, postoperative | Ward nurse | Daily emotional assessment during nursing rounds; structured family participation | All patients |
| Discharge planning | From admission, finalised at discharge | Ward nurse / discharge coordinator | Discharge against standardised criteria; education on home care and follow-up | All patients |

*Note: The intervention was delivered as an integrated, multidisciplinary ERAS-based programme. Because the study was retrospective, per-component adherence was not prospectively recorded; the intended targets above describe the protocol as implemented and should not be interpreted as measured adherence rates.*

## Supplementary Table S2. STROBE checklist for cohort studies

| **STROBE item** | **No.** | **How addressed** | **Location** |
| --- | --- | --- | --- |
| Title and abstract | 1 | Study design in title; structured summary in Abstract | Title; Abstract |
| Background/rationale | 2 | Rationale for evaluating nursing-led ERAS care | Introduction |
| Objectives | 3 | Pre-specified hypothesis stated | Introduction |
| Study design | 4 | Retrospective historical cohort design stated | Methods, Study Design |
| Setting | 5 | Single tertiary centre; recruitment periods 2022 and 2023-2024 | Methods, Study Design |
| Participants | 6 | Eligibility and exclusion criteria; grouping by care period | Methods, Study Population |
| Variables | 7 | Outcomes, exposures, confounders defined | Methods, Outcome Measures |
| Data sources/measurement | 8 | Extraction from electronic records; complication and outcome definitions | Methods, Outcome Measures |
| Bias | 9 | Temporal/historical-control confounding addressed; adjustment methods | Methods; Discussion |
| Study size | 10 | Post-hoc power described; no a priori calculation | Methods, Sample-size considerations |
| Quantitative variables | 11 | Handling of continuous/categorical variables and change from baseline | Methods, Statistical Analysis |
| Statistical methods | 12 | t-test/chi-square, multivariable regression, PSM, IPTW, mixed model, FDR | Methods, Statistical Analysis |
| Participants (results) | 13 | Numbers per group | Results, Baseline |
| Descriptive data | 14 | Baseline characteristics including confounders and baseline labs | Results, Table 1 |
| Outcome data | 15 | Primary and secondary outcomes reported | Results, Tables 2-6 |
| Main results | 16 | Unadjusted and adjusted estimates with 95% CI | Results, Tables 2, 7 |
| Other analyses | 17 | Sensitivity analyses (PSM, IPTW), subgroup by baseline | Results; Table 7 |
| Key results | 18 | Summary with reference to objectives | Discussion |
| Limitations | 19 | Historical control, adherence, instrument, residual confounding | Discussion, Limitations |
| Interpretation | 20 | Cautious, hypothesis-generating interpretation | Discussion; Conclusions |
| Generalisability | 21 | Single-centre limitation noted | Discussion |
| Funding | 22 | Funding statement provided | Funding |
